# Designers of Nature’s Subterranean Abodes: Insights into the Architecture and Utilization of Burrow Systems of Thomas’ Pine Vole, *Microtus thomasi* (Rodentia: Arvicolinae)

**DOI:** 10.3390/life13122276

**Published:** 2023-11-29

**Authors:** Eleni Rekouti, Pavlos Avramidis, Sinos Giokas, Stamatis Vougiouklakis, Sofia Spanou, George P. Mitsainas

**Affiliations:** 1 Section of Animal Biology, Department of Biology, University of Patras, 26504 Patras, Greece; elrekouti@gmail.com (E.R.); sinosg@upatras.gr (S.G.); 2 Department of Geology, University of Patras, 26504 Patras, Greece; p.avramidis@upatras.gr; 3 Department of Material Science, University of Patras, 26504 Patras, Greece; 4 Section of Plant Biology, Department of Biology, University of Patras, 26504 Patras, Greece; saspanou@upatras.gr

**Keywords:** fossorial, burrow structure, subterranean ecology, fractal dimension, soil substrate, soil chemistry

## Abstract

*Microtus thomasi* (Rodentia: Arvicolinae), a fossorial vole endemic to the SW Balkans, uses a variety of substrates but its underground behavior remains poorly understood. This study examines the architecture and utilization of *M. thomasi* burrow systems in NW Peloponnese, Greece. In particular, eight burrow systems were meticulously excavated and studied, with comprehensive measurements taken of key characteristics, including length, depth, soil mounds, and surface openings. Key coordinates were recorded using a differential GPS device for detailed mapping and fractal dimension analysis using the box-counting method was employed to assess burrow system complexity. Soil samples were analyzed for particle size and chemical composition, and vegetation types at each site were identified. We did not find statistically significant correlations between size and complexity of the burrow systems and soil composition, altitude, or specific soil components. On the other hand, we did observe statistically significant differences in tunnel diameter between two burrow systems and in tunnel depth between more. Moreover, our study showed that more than one same-sex individual can occupy a single burrow system and not just an adult male-female pair, that was previously recorded, indicating the need for further study of the social behavior of this vole species. This study provides valuable insights into the underground behavior of *M. thomasi* by providing information on the features of its burrow systems, thus contributing to our understanding of its biology.

## 1. Introduction

*Microtus thomasi* (Rodentia: Arvicolinae) is an endemic vole species of the western Balkan Peninsula. In particular, it occurs in Montenegro, Bosnia and Herzegovina, Albania, and a significant portion of mainland Greece [1,2,3,4,5]. This species occupies a variety of substrates, such as grassland, agricultural fields, and open areas in forests, and has a wide altitudinal range, from sea level up to 2000 m [6].

Thomas’ pine vole has adopted fossorial behavior, primarily conducting its activities underground. However, it occasionally emerges to the surface to search for and consume food [7,8]. As a herbivore, its foraging behavior is strongly influenced by the type and abundance of vegetation in its habitat.

*Microtus thomasi* constructs burrow systems that serve a variety of functions, such as moving, foraging, reproducing, and nurturing of offspring. Living underground provides *M. thomasi* with protection against predators and offers more stable environmental conditions, such as temperature, compared with above-ground habitats [7,9,10,11,12,13,14,15,16,17,18]. For example, as the depth below the ground increases, the temperature tends to become more stable [19], reaching relatively constant values at depths exceeding 50–60 cm [20,21,22,23]. However, despite these advantages, burrow systems do present their inhabitants with some significant shortcomings. An underground-dwelling rodent consumes considerable energy in digging, constructing, expanding and maintaining its burrows [24,25]. The amount of spent energy is influenced by factors such as humidity, and type and hardness of the soil [7,10,25,26,27,28]. Moreover, the tunnels create a unique microclimate, characterized by hypercapnic and hypoxic conditions, which, however, do not appear to harm the animals [7,18,25,27,29,30,31,32].

The degree and characteristics of Thomas’ pine vole’s social behavior are still unknown. However, based on the limited data from a previous study, there are indications that it behaves as a social vole, as evidenced by the presence of male-female pairs and their offspring within a burrow system [33]. Nevertheless, further data are needed to fully comprehend the dynamics and relationships among individuals within the burrow systems of this vole species.

*Microtus thomasi* has attracted significant scientific attention, primarily owing to its extensive chromosomal variability, encompassing both its overall karyotype and sex chromosomes [2,3,4,34,35,36,37,38,39,40]. Nevertheless, only one study has been undertaken, to date, which primarily explored aspects of *M. thomasi*’s biology and behavior [33]. This work aims at expanding our knowledge of this species through a multifaceted approach that includes comprehensive descriptions of burrow architecture and key attributes, digitization of burrow systems for mapping purposes, estimation of burrow complexity, analysis of soil parameters, and statistical analysis of the collected data. It seeks to uncover potential correlations between these factors and the morphology/complexity of *M. thomasi* burrow systems, thus enhancing our understanding of this vole species. Additionally, limited direct behavioral observations were conducted, although these were not part of a systematic behavioral study but were primarily anecdotal in nature.

## 2. Materials and Methods

### 2.1. Field Work and Measurements

**Figure 1:**
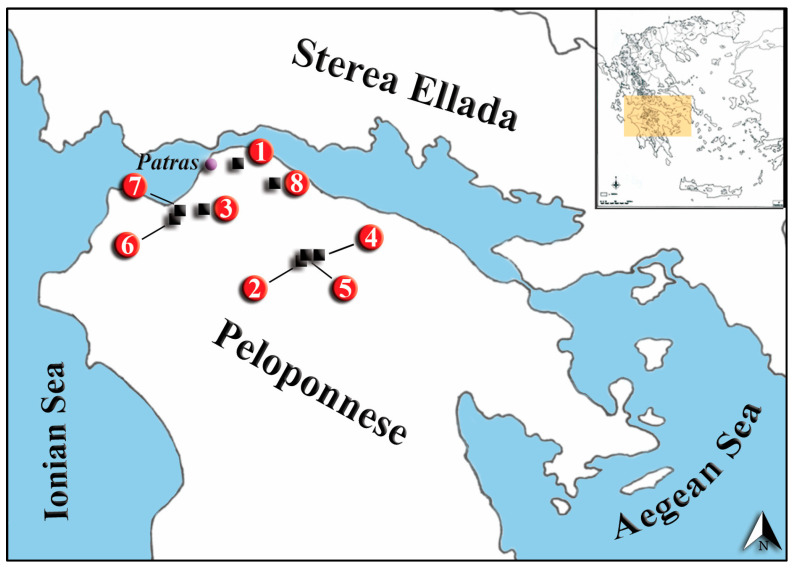
Map of study sites in NW Peloponnese, Greece. Numbers correspond to burrow system localities in Table 1.

The field work was conducted in Achaia Prefecture, located in NW Peloponnese, Greece during two separate time periods (April–September 2016 and April–July 2017). The study sites were carefully chosen to encompass a range of altitudes, ecosystems, and soil types, allowing for a comprehensive assessment of the factors influencing the construction patterns of the species’ burrow systems. A total of eight different localities were selected, spanning an altitude range of 72 to 1624 m a.s.l. (Figure 1, Table 1). In each of these localities, a single burrow system was uncovered for detailed analysis.

The vole presence in each area was determined by identifying the characteristic soil mounds that the species deposits on the surface while digging burrows. The burrow systems were then uncovered gradually by carefully removing the tunnel ceiling, either by hand or using a small shovel. Detailed observations were made for each system, noting features such as the number of nests or food caches. Detailed drawings of the burrow systems were also created by hand on paper to document their structure. The dimensions of each tunnel were determined using a measuring tape. In particular, twenty measurements of tunnel diameter and depth were taken from each burrow system, except for System 3, which, due to its small size, was restricted to ten measurements. In addition, precise coordinates for each burrow system were recorded using a high-resolution differential GPS device. Measurements were made using the Leica GS08 Plus GNSS receiver, which operated without a dedicated base station. Differential position corrections were obtained from the Greek Hellenic Positioning System (HEPOS) via the GSM network using Real-Time Kinematic (RTK) technology. Soil samples, each weighing 50 g, were collected from inside the tunnels of every burrow system for the soil analysis. The number of soil samples per system ranged from two to four, and the locations from which they were collected varied depending on the total length of the central tunnel in each system and the heterogeneity of soil types at each site. This variation aimed at obtaining representative soil parameters for the region.

### 2.2. Map Digitization

The measurements obtained using the differential GPS device were integrated with the recorded features and the drawings of each burrow system. This process enabled the digitization of the burrow system maps using the free and open source QGIS (Ver. 3.28.4) software.

### 2.3. Calculation of Fractal Dimension

A fractal dimension (FD) serves as a natural measure of the complexity of burrow systems [41]. A higher FD value represents a more complex burrow system. To calculate the FD, the digitized maps were processed using the FracLac plug-in of the ImageJ image processing program [42]. The FracLac plug-in utilizes the box-counting method, where a grid is created and the number of squares (N(ε)) required to cover the entire image of a digitized map is counted. Each square has a side length of ε, and the regression slope of logN(ε) plotted against log(1/ε) provides the FD value [43].

### 2.4. Soil Analyses

At the eight study sites, standardized soil sample analyses were conducted, which encompassed various parameters. Particle size analysis was performed using the dry sieving method and the results were classified according to the Folk nomenclature. The determination of total carbon (TC) and total nitrogen (TN) involved the use of a Carlo Erba EA1108 CHNS-O Elemental analyzer. Total organic carbon (TOC) content was estimated using the titration method [44,45,46,47]. This method employed exothermic heating and oxidation of organic carbon with potassium dichromate and concentrated H_2_SO_4_, followed by titration of excess dichromate with 0.5 N ferrous ammonium sulphate solution to a sharp one-drop endpoint. Total phosphorus (TP) was calculated based on the persulfate digestion method specified in APHA 4500-P (2005). Calcium carbonate CaCO_3_ content was determined using the FOG II/Digital soil calcimeter Version 2/2014 (BD INVENTIONS). The calculation of CaCO_3_ (%) relied on measuring the emitted CO_2_ through a modified method [48,49]. Soil color was identified using a Minolta CM-2002 handheld spectrophotometer, and the hue value and chroma were calculated based on the Munsell book of colors. To compare soil characteristics and chemical soil components among the study sites, non-parametric tests (Kruskal-Wallis test and Post-hoc Test Bonferroni) were carried out.

### 2.5. Plant Identification

Identification of plant species found at the study sites was conducted via direct field observation and analysis of photographic material from the sites. Moreover, an attempt was made to identify plant species found in the food caches of *M. thomasi* based on their respective plant parts (dried stems, leaves etc.). Plant species not identified in situ were collected and later processed in the Herbarium of the University of Patras. Plant identification was performed with the help of Flora Europaea [50,51,52,53,54,55], Flora d’ Italia [56], and Flora Hellenica [57,58]; nomenclature was based on the Vascular plants of Greece checklist [59]. Identification of the various vegetation types of the study sites was achieved with direct on-site observation based on the characteristic and diagnostic plant taxa, according to relevant published literature [60,61,62].

### 2.6. Statistical Analyses

Statistical analyses were performed using PAST 4.11 [63]. To compare the diameter and depth of tunnels among the burrow systems, both parametric (ANOVA) and non-parametric tests (Kruskal-Wallis test and Mann-Whitney U-test) with Bonferroni corrections were utilized. Using mean values, both Pearson product-moment and Spearman Rank correlations were conducted to identify potential correlations between the burrow system features (FD and Total length) and the measured environmental parameters. Finally, a Partial Least Squares Regression (PLS Regression) was carried out in order to examine possible relations between the group of the environmental parameters and the group of the factors (FD, Total Length) of the morphology of the burrow systems.

## 3. Results

### 3.1. Field Observations—Burrow Systems Features

The measurements recorded for each uncovered burrow system are presented in Table 2. The total length of the burrow systems ranged from 8.76 m to 87.49 m. Each burrow system appeared to have one central tunnel, ranging in length from 6.03 m to 26.23 m, as well as several side tunnels. Most burrow systems contained two nest chambers, located at a deeper level than the tunnels, and the size of the nest bedding differed between the two chambers. Food cache areas, with numbers ranging from zero to nine, were observed in the systems. However, the food caches were found to be virtually empty, except for System 6, which contained nine filled ones. No latrines were discovered in any of the studied systems. Moreover, half of the studied systems had no open entrances, while the other half had, at most, two. The number of soil mounds counted per system ranged from 10 to 85. Regarding the social behavior of the species, during our field work we observed that three burrow systems (Systems 4, 6 and 8) were occupied at the same time by three or four individuals, indicating that a burrow system of *M. thomasi* can be shared by multiple adult individuals of the same sex. In addition, two females with their offspring inhabited System 5 at the same time, with each female and its offspring occupying a separate nest chamber. Additionally, it was noted that *M. thomasi* individuals will attempt to further hide or abandon their burrow system altogether only under intense disturbance.

### 3.2. Calculation of Fractal Dimension—Map Digitization

**Figure 2:**
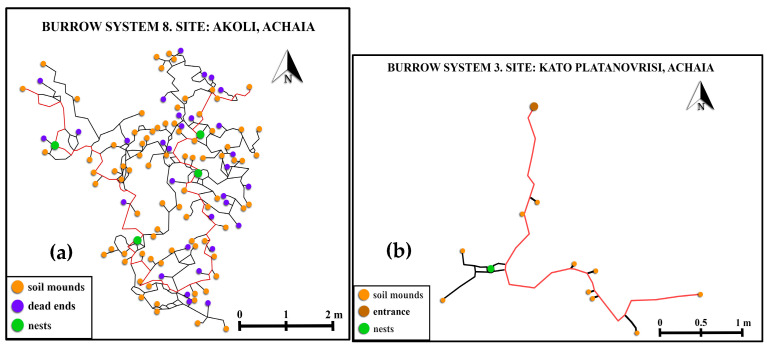
Burrow system (**a**) with the highest FD value (System 8); (**b**) Burrow system with the lowest FD value (System 3). For further details, see Table 2.

The estimated FD values of the burrow systems were found to range from 1.1795 (System 3) to 1.4787 (System 8) (Table 2). These values indicate that System 8 is the most complex one. Figure 2 provides a visual representation of the two burrow systems with the highest and lowest complexity, respectively.

### 3.3. Soil Composition

The results of the soil analyses conducted at the sites with the burrow systems are presented in Table S1. These results revealed significant differences among study sites in terms of soil classification (except for CaCO_3_, possibly due to its low detectability at some sites) and chemical soil components (see Tables S2 and S3). The most consistent difference for almost all the examined soil variables was found at Site 4. Moreover, the percentages of TN and TC of Sites 1 and 2 presented statistically significant differences. However, it is clear that, among the study sites, there are soil samples characterized by a high percentage of gravel and others by a high percentage of sand. Furthermore, it was observed that at certain study sites, the percentage of CaCO_3_ was either extremely low or below the detection limit, which was rather unexpected for areas in the Mediterranean region, which is known to have soils rich in CaCO_3_.

### 3.4. Plant Identification

The vegetation type at each study site is presented at Table 3.

Plant species identified at each study site from direct field observations, photographic material, and collected dry samples, are presented in Table S4. In areas where the burrow systems were established, most plant species belonged to families Asteraceae and Poaceae, which are common in Mediterranean vegetation. The plant parts that were found in the food caches, most of them in the form of small stem parts, were difficult to identify to genus or species level due to the lack of taxonomical features. However, it was possible to identify them as mainly belonging to family Poaceae (grasses).

### 3.5. Statistical Analyses

#### 3.5.1. Tunnel Diameter and Depth

**Figure 3:**
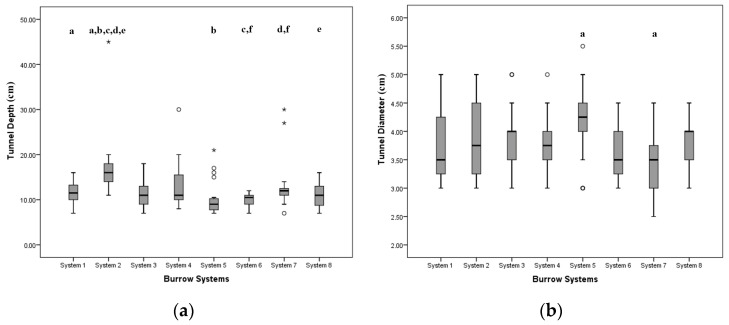
Box plots of the variance (**a**) of tunnel depth of burrow systems; (**b**) of diameter of burrow systems. Small circles: potential outliers; asterisks: extreme outliers; Letters a-f indicate the pairs of burrow systems that differed significantly.

Figure 3 demonstrates the variation in tunnel depth and diameter across all studied burrow systems. Levene’s test for homogeneity of variance was non-significant both for tunnel depth and diameter (*p* > 0.1). However, the Kolmogorov-Smirnov statistical test indicated that the sample did not follow a normal distribution (*p* < 0.05), necessitating the use of the non-parametric Kruskal-Wallis test. The results of this test (*p* < 0.05) revealed significant differences in tunnel depth and diameter among the studied systems (Table S5), which led to the implementation of post-hoc pairwise comparison by Mann-Whitney U-test with a Bonferroni correction. This further demonstrated that, with regard to tunnel diameter, System 5 differed significantly (*p* < 0.05) from System 7. In terms of tunnel depth, System 2 differed from most other systems (*p* < 0.05), except for Systems 3 and 4. Furthermore, System 6 exhibited significant difference from System 7 (*p* < 0.05). There were no significant differences observed in the other system pairs (Table S6).

#### 3.5.2. Correlation of Burrow System Attributes with Fractal Dimension and Total Length

No significant correlations were found between burrow system attributes with Fractal Dimension and Total Length (Table S7). Similarly, the Partial Least Squares Regression did not reveal any significant relations (Squared Covariance 11.602%, *p* = 0.701) between the group of the environmental parameters and the group of the factors (FD, Total Length) describing the morphology of the burrow systems.

## 4. Discussion

We conducted a comprehensive investigation into the architecture, attributes, and complexity of the burrow systems created by Thomas’ pine vole (*Microtus thomasi*) aiming at enriching the existing data on its fossorial behavior. To achieve this, we carefully selected study sites within the distribution range of the species in NW Peloponnese. Our site selection encompassed various altitudes, habitats, and soil types, allowing us to examine the potential influence of these factors on the construction pattern of the species’ burrow systems.

The burrow systems excavated by Thomas’ pine vole consisted of a central tunnel and several side tunnels, which seemed to differ in diameter and depth among specific systems. According to our literature review, this observation has not been recorded in previous relevant studies of the species. The systems primarily contained two chambers, utilized as nests, and also included multiple food caches and numerous dead ends, which refer to the extremities of the system that did not lead to soil mounds or surface openings. It is worth mentioning that the construction of the nest chambers at a deeper level than the tunnels aligns with findings from previous studies. This pattern suggests a preference among many subterranean species to construct their nest chambers at deeper levels compared with foraging tunnels, a behavior that serves to minimize thermoregulation activity, maximize protection from predators, and ensure the structural integrity of the nest [22,32,64,65,66].

During the summer period and under high temperatures, many subterranean species adopt a strategy of opening multiple entrances that allow their burrow systems to connect with the open air aboveground. This behavior serves to enhance ventilation and aid in thermoregulation within the tunnels and chambers [20,32,67]. In our study, we detected a similar behavior in *M. thomasi*, which, based on personal observations, appears to seal off all entrances during the winter period but to open a few during the summer. However, the number of open entrances recorded in our study ranged from 0 to 2, which is, by comparison, lower than the corresponding number reported for other subterranean vole taxa, such as *Microtus ochrogaster* [68,69,70], *M. lusitanicus* [71], *Otomys sloggetti robertsi*, *Parotomys brantsii*, *P. littledalei* [14], and *Ctenomys* sp. [72].

As mentioned previously, the majority of excavated systems were characterized by the presence of several food caches. However, due to the vacancy of these caches in most cases, it was occasionally challenging to distinguish them from dead ends. This observation suggests that *M. thomasi* likely consumes its food immediately after harvesting, or shortly thereafter. Additionally, in areas with ample food availability, the need to store food diminishes [64,68,71,73]. In this context, the discovery of nine filled food caches in System 6 stands out as an interesting exception, which could be related to the fact that this system was established in an area with dense presence of other burrow systems (as determined by the arrangement of soil mounds on the ground), indicating, perhaps, a high local vole population density. It would be interesting to further survey if this vole species, when faced with intense competition for food resources, invests time and energy to create numerous food caches in its burrow systems, to be kept filled with food, in an effort to outcompete their numerous nearby rivals. This would be in agreement with another study conducted on *Peromyscus leucopus* [74], which highlighted that the quantity of food stored in underground caches and storage duration depend on the presence of competitors and the availability of food resources in the surrounding area.

Regarding the complexity of the studied burrow systems, in terms of Fractal Dimension, the measured value range in this study (1.18–1.48) is comparable to those estimated for several subterranean taxa, such as *Heterocephalus glaber* [75,76], *Cryptomys hottentotus* [77], *Heliophobius argenteocinereus* [78], *Spalax galili* [27], *Microtus lusitanicus* [71], and *Fukomys* sp. [41]. However, it is lower than that of *Fukomys anselli* [79].

Concerning the management of excess soil, usually, subterranean rodents either discard the excavated soil on the ground surface, creating soil mounds, typical for each species, or push the soil inside old tunnel sections, in order to seal them [18,80]. The second option is more energy-efficient compared with pushing soil upwards to the ground surface [80,81]. Furthermore, by sealing deserted tunnel sections, voles protect themselves from predators, such as reptiles, since it becomes difficult and energy-consuming to patrol a very extended system, but easier to isolate parts of it [18,72]. In our work, as well as soil mounds, we also noticed sealed tunnels in every uncovered system. Therefore, *M. thomasi* seems to routinely use this strategy too, regarding excess soil disposal.

The burrows of subterranean rodents typically have limited gas ventilation, causing low O_2_ and high CO_2_ concentrations, leading to hypoxia and hypercapnia, respectively [7,17,20,29,32,82]. These gas conditions in the tunnel microclimate induce significant stress on subterranean rodents [30,83,84,85,86,87], including a decrease in the metabolic rate of inhabitants, thereby reducing their spare energy, which is crucial to their digging ability in the burrows. Furthermore, high or low soil porosity directly affect O_2_ and CO_2_ levels in ground layers. In this sense it was expected that *M. thomasi* would prefer to create its burrow systems at sites with high soil porosity, as the quantity of O_2_ would be higher. However, our results indicated that the creation, complexity, and total length of the burrow systems of *M. thomasi* are not influenced by soil composition.

Previous studies have suggested that the number of inhabitants in a burrow system is positively correlated to its complexity and size. The rationale behind this correlation is that a larger number of individuals can explore the area more extensively by moving underground [41,78]. It would be interesting to survey whether a similar behavior is also adopted by *M. thomasi*, and, moreover, whether an increase in the density of different burrow systems in a region would negatively affect the total length of individual burrow systems, due to limited available space. Such findings would suggest a relationship between population density, spatial distribution of burrow systems and burrow complexity.

Finally, in the only other relevant study on *M. thomasi* populations in Greece [33], it was observed that a single burrow system would be occupied by a male-female pair and their offspring. In our study, it was recorded that multiple adult individuals of the same sex can inhabit a burrow system at the same time, which constitutes a new observation. Moreover, the reaction of hiding or abandoning their system only under intense disturbance is similar to observations made in that previous study from Greece.

### Study Limitations

Based on our findings, it is evident that *M. thomasi* exhibits interesting social dynamics; however, additional research is required to fully understand the degree of sociality in this vole species. Furthermore, the inability to detect statistically significant correlations among parameters of burrow architecture and environmental variables does not necessarily mean the absence of environmental influences on burrow architecture. In fact, it could be just a statistical artifact due to the relatively small sample size. Thus, conducting future studies that encompass a larger number of burrow systems across different areas within the species’ distribution will provide a more comprehensive understanding of their burrow architecture, complexity, utilization, and the factors influencing them.

## 5. Conclusions

This study has yielded valuable insights into the architecture and utilization of burrow systems of Thomas’ pine vole (*Microtus thomasi*). Notably, our statistical analysis indicates that the dimensions and complexity of the burrow systems do not appear to be significantly influenced by soil composition, altitude, or specific chemical soil components. Furthermore, the discovery that burrow system occupancy by adults appears to be more intricate than a simple male-female pair arrangement motivates further investigation into the social behavior of this vole species. Overall, our research contributes to our understanding of the subterranean behavior of *M. thomasi* by providing a detailed description of the features of its burrow systems, which, in turn, advances our knowledge of its biology. Moreover, this information has broader implications. It can be applied to the concept of ecosystem engineering by small mammals, since understanding how small mammals modify their habitats through burrow system construction can help ecologists comprehend their ecological roles in shaping local environments. For *M. thomasi*, further studies are essential to fully comprehend the dynamics and relationships among individuals within the burrow systems.

## Figures and Tables

**Table 1:** Geographical information on the localities of the studied burrow systems (numbering as in Figure 1).

Locality	System	Coordinates (Lat/Long)	Altitude (m)
Argira	1	38.282464, 21.860464	489
Ano Loussoi	2	38.000889, 22.137148	1112
Kato Platanovrisi	3	38.139761, 21.734952	272
Mt. Aroania ski resort	4	38.013551, 22.190700	1624
Agios Nikolaos	5	38.008342, 22.150155	1235
Kalamaki	6	38.104195, 21.626795	73
Mireika	7	38.128207, 21.643241	72
Akoli	8	38.226368, 22.003262	305

**Table 2:** Measurements and estimates of burrow systems of *M. thomasi*. Syst.: Burrow system; FD: Fractal dimension value, based on the box-counting method. Bold: the highest value of each measurement.

Measurements	Syst.1	Syst.2	Syst.3	Syst.4	Syst.5	Syst.6	Syst.7	Syst.8
Total Length (m)	57.16	59.83	8.76	77.64	24.49	42.61	24.13	**87.49**
Length of central tunnel (m)	22.02	**26.23**	6.03	22.62	8.63	12.15	8.21	19.28
Average tunnels diameter (cm)	3.775 ±0.31	3.800 ±0.30	3.850 ±0.29	3.775 ±0.27	**4.175** **±0.32**	3.600 ±0.21	3.425 ±0.23	3.800 ±0.23
Average tunnels depth (cm)	11.625 ±1.19	**17.050** **±3.33**	11.300 ±2.29	13.150 ±2.36	10.325 ±1.80	9.950 ±0.65	13.025 ±2.59	11.075 ±1.33
Nests	2	2	1	2	2	2	1	**4**
Food caches	7	2	0	3	0	**9**	6	0
Open entrances	0	**2**	1	0	0	**2**	1	0
Soil mounds	23	30	10	49	38	84	38	**85**
Dead ends	24	17	0	20	7	7	3	**32**
FD	1.2283	1.2735	1.1795	1.3607	1.2677	1.3722	1.3036	**1.4787**

**Table 3:** Description of vegetation at each study site.

Study Sites	Vegetation Type
System 1	Fallow field
System 2	Fallow field
System 3	Fallow field
System 4	*Abies cephalonica* forest openings
System 5	Fallow field
System 6	Olive grove with dense herbaceous understory of therophytes
System 7	Olive grove with removed understory by grass trimmer
System 8	Olive grove with removed understory by grass trimmer

## Data Availability

The data presented in this study are available in Table 1 and Table 2 within the article and in the submitted Supplementary Materials (Tables S1–S4).

## Supplementary Materials

The following supporting information can be downloaded at: https://www.mdpi.com/article/10.3390/life13122276/s1, Table S1: Results of soil analysis of the samples from the burrow systems; Table S2: Results of the non-parametric Kruskal-Wallis test comparing soil variables of study sites; Table S3: Results of the non-parametric Post-hoc Test Bonferroni, comparing soil variables among study sites (only the pairs of sites with statistically significant differences are displayed); Table S4: Results of plant identification from each study site. Table S5: Results of the non-parametric Kruskal-Wallis test comparing tunnel diameter and tunnel depth of burrow systems; Table S6: Results of the non-parametric Mann-Whitney U-test, with Bonferroni corrected *p*-values, comparing (a) tunnel diameter and (b) tunnel depth of the burrow systems (only the burrow system pairs with statistically significant differences are displayed); Table S7: Correlation ((a) Pearson product-moment correlation and (b) Spearman’s test) of environmental attributes with Fractal Dimension (FD) and Total Length of burrow systems.
