# Supplementary material for: Designers of Nature’s Subterranean Abodes: Insights into the Architecture and Utilization of Burrow Systems of Thomas’ Pine Vole, Microtus thomasi (Rodentia: Arvicolinae)
Source: Life (Basel). 2023 Nov 29;13(12):2276. doi: 10.3390/life13122276 (PMC10744952; doi:10.3390/life13122276)
Supplement: Supplementary file 1 [file life-13-02276-s001.zip › life-2636576-supplementary.pdf]

Supplementary material for Rekouti et al. *M. thomasi* burrow system study

Table S1. Results of soil analysis of the samples from the burrow systems.

		% Gravel	% Sand	% Silt	% Clay	% $CaCO_3$	% TOC	TP (mg/L)	% TN	% TC	Soil color	Soil Type (according to Triangular diagram of Folk)
Syst. 1	Sample 1	9.03	44.41	28.69	17.87	< DL	1.06	2.28	0.16	1.34	Pale Brown (10YR 6/3)	Gravelly Mud
	Sample 2	8.26	45.70	29.74	16.30	< DL	0.95	2.04	0.17	1.55	Pale Brown (10YR 6/3)	Gravelly Mud
	Sample 3	3.92	52.37	29.24	14.47	< DL	0.97	3.24	0.15	1.42	Pale Brown (10YR 6/3)	Slightly Gravelly Muddy Sand
Syst. 2	Sample 1	47.20	31.67	10.80	10.34	11.30	3.64	4.28	0.57	6.74	Brown (7.5YR 4/4)	Muddy Sandy Gravel
	Sample 2	38.18	32.59	14.90	14.34	3.90	3.91	5.42	0.59	5.79	Brown (7.5YR 4/4)	Muddy Sandy Gravel
	Sample 3	29.60	41.10	14.98	14.31	3.20	5.04	5.49	0.68	7.51	Brown (7.5YR 4/3)	Gravelly Muddy Sand
	Sample 4	49.28	24.90	11.50	14.33	4.30	3.25	6.52	0.43	4.58	Brown (7.5YR 4/3)	Muddy Gravel
Syst. 3	Sample 1	4.30	42.06	34.44	19.20	1.00	1.53	1.06	0.21	2.39	Light Yellowish Brown (10YR 6/4)	Slightly Gravelly Sandy Mud
	Sample 2	1.90	36.24	37.90	23.95	3.40	1.40	1.05	0.24	2.37	Light Yellowish Brown (10YR 6/4)	Slightly Gravelly Sandy Mud
Syst. 4	Sample 1	0.94	28.85	40.85	29.36	2.30	3.97	2.75	0.43	5.56	Brown (7.5YR 5/3)	Slightly Gravelly Sandy Mud
	Sample 2	0.74	30.86	37.12	31.28	2.50	4.07	2.83	0.44	5.70	Brown (7.5YR 5/3)	Slightly Gravelly Sandy Mud
	Sample 3	0.27	28.08	37.66	33.99	2.30	3.72	2.68	0.40	5.32	Brown (7.5YR 4/3)	Slightly Gravelly Sandy Mud
	Sample 4	1.33	22.95	42.26	33.46	10.60	3.86	2.51	0.47	8.03	Brown (7.5YR 5/3)	Slightly Gravelly Sandy Mud
Syst. 5	Sample 1	13.62	44.54	24.48	17.36	0.50	2.14	3.96	0.31	2.96	Reddish Brown (5YR 4/4)	Gravelly Muddy Sand
	Sample 2	14.73	43.82	20.92	20.52	< DL	1.85	2.79	0.27	2.77	Reddish Brown (5YR 4/4)	Gravelly Muddy Sand

Syst. 6	Sample 1	9.86	40.48	30.34	19.32	< DL	1.07	1.49	0.25	2.57	Yellowish Brown (10YR 5/4)	Gravelly Mud
	Sample 2	11.35	42.90	27.91	17.84	< DL	0.96	0.96	0.18	1.65	Yellowish Brown (10YR 5/4)	Gravelly Mud
Syst. 7	Sample 1	6.13	45.03	24.19	24.65	1.20	1.27	0.72	0.25	2.30	Brown (10YR 5/3)	Gravelly Mud
	Sample 2	7.27	37.68	31.75	23.30	0.90	1.37	0.78	0.26	2.37	Brown (10YR 5/3)	Gravelly Mud
Syst. 8	Sample 1	44.95	30.94	11.93	12.18	< DL	1,40	1.10	0.18	2.00	Brown (7.5YR 5/4)	Muddy Sandy Gravel
	Sample 2	58.84	22.42	10.34	8.40	< DL	1.41	0.89	0.18	1.72	Brown (7.5YR 5/4)	Muddy Sandy Gravel
	Sample 3	48.76	28.99	11.55	10.70	< DL	1.36	1.21	0.16	1.46	Brown (7.5YR 5/4)	Muddy Sandy Gravel

Table S2. Results of the non-parametric Kruskal-Wallis test comparing soil variables of study sites.

**Parameters**	**Kruskal-Wallis (p-value)**
% gravel	19.9269 (0.006)
% sand	17.2964 (0.016)
% silt	19.1818 (0.008)
% clay	19.5929 (0.007)
% $CaCO_3$	19.1164 (0.008)
% TOC	19.5731 (0.007)
TP (mg/L)	19.2352 (0.007)
% TN	19.7826 (0.006)
% TC	19.0523 (0.008)

Table S3. Results of the non-parametric Post-hoc Test Bonferroni, comparing soil variables among study sites (only the pairs of sites with statistically significant differences are displayed).

**Pairs of sites**	**Difference**	**+/- Limits**
**a) Gravel**		
System 2 – System 4	15.75	14.34
System 4 – System 8	-17.50	15.49
**b) Sand**		
System 1 – System 4	15.83	15.49
**c) Silt**		
System 2 – System 4	-15.50	14.34
System 4 – System 8	16.67	15.49
**d) Clay**		
System 2 – System 4	-15.50	14.34
System 4 – System 8	17.83	15.49
**e) $CaCO_3$**		
-	-	-
**f) TOC**		
System 1 – System 4	-16.33	15.49
**g) TP**		
System 2 – System 7	19.00	17.57
**h) TN**		
System 1 – System 2	-17.67	15.49
**i) TC**		
System 1 – System 2	-16.42	15.49
System 1 – System 4	-15.92	15.49

Table S4. Results of plant identification from each studied site.

Studied sites	Plant species of the area outside the tunnels	Plant species found in food caches or tunnels
**System 1**	• *Anagallis arvensis* L.<br>• *Capsella bursa-pastoris* (L.) Medik.<br>• *Carduus pycnocephalus* L.<br>• *Hordeum murinum* L.<br>• *Hypochaeris cretensis* (L.) Bory & Chaub.<br>• *Malva sylvestris* L.<br>• *Helminthotheca echioides* (L.) Holub<br>• *Sinapis arvensis* L.<br>• *Sonchus asper* (L.) Hill<br>• *Taraxacum* sp.	• *Tetragonolobus purpureus* Moench<br>• Several species of Poaceae family
**System 2**	• *Aegilops* sp.<br>• *Allium* sp.<br>• *Avena barbata* Link<br>• *Carduus pycnocephalus* L.<br>• *Convolvulus althaeoides* L.<br>• *Crepis rubra* L.<br>• *Geranium molle* L.<br>• *Lotus* sp.<br>• *Myosotis* sp.<br>• *Smyrnium olusatrum* L.<br>• *Tordylium apulum* L.<br>• *Tragopogon porrifolius* L.<br>• *Verbascum* sp.<br>• *Veronica arvensis* L.<br>• *Vicia* sp.<br>• *Vicia villosa* Roth	• *Ornithogalum* sp.
**System 3**	• Species of Poaceae family (i.e. *Bromus* sp.)<br>• Species of Asteraceae family	-
**System 4**	• *Abies cephalonica* Loudon<br>• *Aethionema* sp.<br>• *Alkanna tinctoria* Tausch	-

	• *Anemone apennina* subsp. *blanta* (Schott & Kotschy)<br>• *Arum* sp.<br>• *Astracantha* sp.<br>• *Ballota acetabulosa* (L.) Benth.<br>• *Bellis annua* L.<br>• *Cruciata taurica* (Willd.) Ehrend.<br>• *Doronicum orientale* Hoffm.<br>• *Hordeum* sp.<br>• *Lamium moschatum* Mill.<br>• *Lepidium* sp.<br>• *Lagoecia cuminoides* L.<br>• *Malcolmia* sp.<br>• *Muscari neglectum* Ten.<br>• *Myosotis* sp.<br>• *Tordylium apulum* L.<br>• *Tulipa australis* Link<br>• *Urtica dioica* L.<br>• *Verbascum* sp.<br>• *Viola aetolica* Boiss. & Heldr.	
**System 5**	• *Abies cephalonica* Loudon<br>• *Anthylis vulneraria* L.<br>• *Asyneuma* sp.<br>• *Campanula* sp.<br>• *Helianthemum salicifolium* (L.) Mill.<br>• *Juniperus oxydercus* L.<br>• *Knautia integrifolia* (L.) Bertol.<br>• *Medicago lupulina* L.<br>• *Onosma erecta* Sm.<br>• *Quercus coccifera* L.<br>• *Silene colorata* Poir.<br>• *Tordylium officinale* L.<br>• *Trifolium stellatum* L.<br>• Species of Poaceae family (i.e. *Bromus* sp., *Aegilops* sp., *Avena* sp.)	-

	• Species of Asteraceae family (i.e. *Crepis* sp., *Centaurea* sp., *Anthemis* sp., *Leontodon crispus* Vill.)<br>• Species of Cruciferae family<br>• Species of Lamiaceae family (i.e. *Salvia* sp., *Acinos alpinus* (L.) Moench)	
**System 6**	• *Anagallis arvensis* L.<br>• *Anthemis arvensis* L.<br>• *Brachypodium* sp.<br>• *Bromus madritensis* L.<br>• *Carduus pycnocephalus* L.<br>• *Echium angustifolium* Mill.<br>• *Silene gallica* L.<br>• *Silybum marianum* (L.) Gaertn.<br>• *Trifolium campestre* Schreb.	• Species of Poaceae family (difficult to further determine)
**System 7**	• *Asparagus acutifolius* L.<br>• *Foeniculum vulgare* Mill.<br>• *Malva punctata* (All.) Alef.<br>• *Scolymus hispanicus* L.<br>• *Verbascum sinuatum* L.<br>• Species of Poaceae family (i.e. *Bromus* sp.)<br>• Species of Asteraceae family<br>• Species of Apiaceae family (i.e. *Tordylium apulum*)	-
**System 8**	• *Olea europaea* L.	-

Table S5. Results of the non-parametric Kruskal-Wallis test comparing tunnel diameter and tunnel depth of burrow systems.

**Parameters**	**Kruskal-Wallis (p-value)**
Tunnel diameter	40.943 (0.000)
Tunnel Depth	16.346 (0.022)

Table S6. Results of the non-parametric Mann-Whitney U-test, with Bonferroni corrected p-values, comparing a) tunnel diameter and b) tunnel depth of the burrow systems (only the burrow system pairs with statistically significant differences are displayed).

**System pairs**	**Mann-Whitney (corrected p-value with Bonferroni)**
**a) Tunnel diameter**	
System 5 – System 7	76.50 (0.018)
**b) Tunnel depth**	
System 1 – System 2	57.00 (0.003)
System 2 – System 5	50.00 (0.001)
System 2 – System 6	11.00 (0.000)
System 2 – System 7	75.00 (0.019)
System 2 – System 8	51.00 (0.002)
System 6 – System 7	83.00 (0.042)

Table S7. Correlation ((a) Pearson product-moment correlation and (b) Spearman's test) of environmental attributes with Fractal Dimension (FD) and Total Length of burrow systems.

(a) Pearson product-moment correlation				
**Variables**	**FD**		**Total Length**	
	**r**	**p**	**r**	**p**
Altitude	-0.052	0.903	0.324	0.433
% Gravel	0.524	0.183	0.538	0.169
% Sand	-0.621	0.101	-0.676	0.066
% Silt	-0.394	0.335	-0.356	0.387
% Clay	-0.168	0.692	-0.201	0.633
$CaCO_3$	-0.178	0.673	0.196	0.643
TOC	0.004	0.993	0.358	0.384
TP	-0.249	0.551	0.221	0.599
TN	-0.082	0.848	0.208	0.621
TC	-0.003	0.995	0.311	0.454
**(b) Spearman's test**				
**Variables**	**FD**		**Total Length**	
	**r**	**p**	**r**	**p**
Altitude	-0.119	0.793	0.500	0.216
% Gravel	0.333	0.409	0.405	0.313
% Sand	-0.524	0.197	-0.548	0.171
% Silt	-0.357	0.389	-0.286	0.501
% Clay	-0.190	0.665	-0.476	0.243
$CaCO_3$	-0.268	0.528	-0.073	0.887
TOC	-0.119	0.793	0.071	0.861
TP	-0.095	0.840	0.429	0.283
TN	-0.048	0.935	0.000	1.000
TC	-0.143	0.752	-0.024	0.977
